# What’s Behind the Spread of White Syndrome in Great Barrier Reef Corals?

**DOI:** 10.1371/journal.pbio.0050164

**Published:** 2007-05-08

**Authors:** Mary Hoff

Coral reefs, among Earth’s richest ecosystems, traditionally teem with an abundance of life. The tiny corals that form the foundation of the community are microcosms themselves, sheltering single-celled algae that feed the community with energy from the sun. The three-dimensional physical space they create with their skeletons supports an astounding array of other animals, from sponges to sharks.

But in recent years, corals have been dying in droves. Scientists suspect a variety of factors, ranging from accidental damage from fishing activity to the effects of polluted runoff from land. One threat that appears to be growing dramatically in Australia’s famed Great Barrier Reef is white syndrome, a mystery-riddled disease that is spreading rapidly, leaving stripes of dead corals like ribbons of death in its wake. But why?

Global warming seems a likely suspect for several reasons. Past epidemiological studies across a broad range of life forms have shown that stress—including the stress of changing environmental conditions—often increases disease susceptibility. As temperatures rise, pathogens can reproduce more quickly. The fact that coral diseases seem to spread faster in summer also provides support for the notion that warmer temperatures may be involved.

In hope of improving understanding of the spread of white syndrome, John Bruno, Amy Melendy, and colleagues conducted a regional-scale longitudinal study of the hypothesized link between warm temperature deviations and the presence of white syndrome, considering the density of coral cover as an additional variable of interest.

To quantify temperature fluctuations, the researchers used a high-resolution dataset on ocean surface temperature provided by the United States National Oceanic and Atmospheric Association and the University of Miami. They used the dataset to calculate weekly sea surface temperature anomalies (WSSTAs)—instances in which temperature was higher by 1°C or more from mean records for that week—for 48 reefs within the Great Barrier Reef. To evaluate the extent of white syndrome and coral cover, the researchers used data collected by the Australian Institute of Marine Science Long-term Monitoring Program on 48 reefs from a 1,500-kilometer stretch of the Great Barrier Reef from 1998 to 2004 at a depth of 6 to 9 meters. Divers counted the number of infected colonies on each reef. Coral cover, the amount of the bottom with living corals, was measured from videos taken of the reefs.

**Figure:**
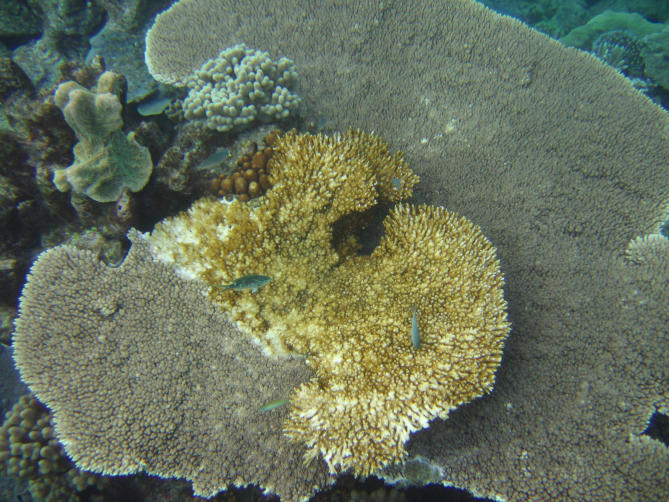
A coral colony with white syndrome from the Great Barrier Reef. (Image: Australian Institute of Marine Science Long-term Monitoring Program)

The researchers then evaluated the relationship between the occurrence of white syndrome and three variables: number of WSSTAs occurring during the previous 52 weeks, coral cover, and the interaction between the two. They found that the third variable showed a statistically significant correlation with number of white syndrome cases, indicating that the presence of both conditions—temperature anomalies and high coral cover—creates the conditions in which white syndrome outbreaks are most likely to occur. In other words, WSSTAs were a necessary but not sufficient condition for white syndrome outbreaks, whereas the combination of heat stress and a dense colony was deadly.

What does this mean for corals and the ecosystem they support? If global warming increases the incidence of warm temperature anomalies in tropical oceans the years ahead, these results suggest that corals in high-cover areas will be increasingly vulnerable to white disease. If the effect is large enough, the tightly woven web of life within coral reefs could begin to unravel, potentially transforming habitats that were once among the planet’s richest ecosystems into underwater wastelands.

This strong evidence for a link among a warming ocean, coral density, and white syndrome provides a rich foundation for further work to understand the spread of coral disease in the Great Barrier Reef. It also provides valuable insights into marine epidemiology that could be of much value in investigating and potentially mitigating other devastating global-warming–related disease outbreaks in the world’s vast and vulnerable oceans.

